# Risk of diabetic retinopathy and retinal neurodegeneration in individuals with type 2 diabetes: Beichen Eye Study

**DOI:** 10.3389/fendo.2023.1098638

**Published:** 2023-05-03

**Authors:** Zhengwei Yang, Qingyan Liu, Dejia Wen, Zihao Yu, Chuanzhen Zheng, Fei Gao, Chen Chen, Liying Hu, Yu Shi, Xiuqing Zhu, Juping Liu, Yan Shao, Xiaorong Li

**Affiliations:** Tianjin Key Laboratory of Retinal Functions and Diseases, Tianjin Branch of National Clinical Research Center for Ocular Disease, Eye Institute and School of Optometry, Tianjin Medical University Eye Hospital, Tianjin, China

**Keywords:** risk factors, type 2 diabetes mellitus, diabetic retinopathy, retinal neurodegeneration, macular ganglion cell-inner plexiform layer, risk of diabetic retinopathy and retinal neurodegeneration

## Abstract

**Objective:**

Our aim was to evaluate associations of different risk factors with odds of diabetic retinopathy (DR) diagnosis and retinal neurodegeneration represented by macular ganglion cell-inner plexiform layer (mGCIPL).

**Methods:**

This cross-sectional study analyzed data from individuals aged over 50 years examined between June 2020 and February 2022 in the community-based Beichen Eye Study on ocular diseases. Baseline characteristics included demographic data, cardiometabolic risk factors, laboratory findings, and medications at enrollment. Retinal thickness in both eyes of all participants was measured automatically *via* optical coherence tomography. Risk factors associated with DR status were investigated using multivariable logistic regression. Multivariable linear regression analysis was performed to explore associations of potential risk factors with mGCIPL thickness.

**Results:**

Among the 5037 included participants with a mean (standard deviation, SD) age of 62.6 (6.7) years (3258 women [64.6%]), 4018 (79.8%) were control individuals, 835 (16.6%) were diabetic individuals with no DR, and 184 (3.7%) were diabetic individuals with DR. The risk factors significantly associated with DR status were family history of diabetes (odds ratio [OR], 4.09 [95% CI, 2.44-6.85]), fasting plasma glucose (OR, 5.88 [95% CI, 4.66-7.43]), and statins (OR, 2.13 [95% CI, 1.03-4.43]) relative to the control individuals. Compared with the no DR, diabetes duration (OR, 1.17 [95% CI, 1.13-1.22]), hypertension (OR, 1.60 [95% CI, 1.26-2.45]), and glycated hemoglobin A1C (HbA1c) (OR, 1.27 [95% CI, 1.00-1.59]) were significantly correlated with DR status. Furthermore, age (adjusted β = -0.19 [95% CI, -0.25 to -0.13] μm; *P* < 0.001), cardiovascular events (adjusted β = -0.95 [95% CI, -1.78 to -0.12] μm; *P* = 0.03), and axial length (adjusted β = -0.82 [95% CI, -1.29 to -0.35] μm; *P* = 0.001) were associated with mGCIPL thinning in diabetic individuals with no DR.

**Conclusion:**

Multiple risk factors were associated with higher odds of DR development and lower mGCIPL thickness in our study. Risk factors affecting DR status varied among the different study populations. Age, cardiovascular events, and axial length were identified as potential risk factors for consideration in relation to retinal neurodegeneration in diabetic patients.

## 1 Introduction

Diabetic retinopathy (DR), the most common microvascular complication of diabetes, remains a major cause of preventable vision loss worldwide in the working population (1). Type 2 diabetes mellitus (T2DM) is widely recognized as a causative factor of retinal neuropathy and vasculopathy (2). Moreover, retinal neurodegeneration that occurs during the progression of diabetes is reported to contribute to microvascular abnormalities (3). Accumulating evidences in recent years suggests that retinal neurodegeneration precedes the appearance of detectable microvasculopathy (4, 5). Histologic findings further indicate that retinal neurodegeneration is involved in neural apoptosis, loss of ganglion cell bodies, reactive gliosis, and reduction of the inner retinal layers, which results in thinning of the macular ganglion cell-inner plexiform layer (mGCIPL) (6). Optical coherence tomography (OCT) is a commonly used technique to assess the thickness of mGCIPL (7).

Various risk factors play distinct roles in the onset and development of DR (8, 9). Based on collective demographic, epidemiological, and laboratory data, multiple potential risk factors have been identified, including gender, hypertension, glycated hemoglobin A1c (HbA1c), hyperlipidemia, and diabetes duration (10). Additionally, body mass index (BMI) and dyslipidemia are also significant predictors of DR development. However, the consistency and strength of the several risk factors vary significantly (11–13). For instance, high HbA1c levels may account for only approximately 10% of the risk of DR (10), and the combined risk of hypertension and dyslipidemia may be less than 10% in some studies (14), indicating the possibility that additional unknown risk factors contribute to the onset and progression of DR (15).

Retinal neurodegeneration, speculated to be a useful predictor of DR progression, is potentially affected by multiple risk factors (16). The risk factors associated with neurodegeneration vary widely across different reports. Data from the UK Biobank suggested that BMI is negatively correlated with total retinal thickness, mGCIPL thickness, and retinal nerve fiber layer (RNFL) thickness (17, 18). In two earlier studies, age, sex, BMI, higher estimated glomerular filtration rate, high alcohol intake, and refractive error were shown to be associated with thinning of mGCIPL in the general population (19, 20). Another cross-sectional investigation demonstrated correlations of hypertension, statin medication, autonomic nerve function and peripheral nerve conduction with mGCIPL thickness in patients with DR (21). However, few clinical studies have simultaneously investigated the associations of various risk factors with DR status and retinal neurodegeneration. Demographic, clinical, cardiometabolic, laboratory, and treatment factors may emerge as important predisposing factors in DR formation and retinal neurodegeneration.

The main aim of this study was to investigate associations of multiple risk factors with DR diagnosis and mGCIPL thickness in urbanized areas of Tianjin, including four towns and 12 villages, during the coronavirus pandemic. Elucidation of the specific effects of risk factors could aid in prevention and management of DR, thereby improving visual outcomes and patient quality of life.

## 2 Methods

### 2.1 Study population

The Beichen Eye Study is a cross-sectional investigation conducted between June 2020 and February 2022 in Northern China, involving four towns and 12 villages from urbanized areas of the Beicen District located north of Tianjin. This population-based study was performed on adults aged 50 years and over using a standardized protocol. Communities were selected using a multi-stage random sampling procedure. This study was approved by the Ethical Committee of Tianjin Medical University Eye Hospital, and performed according to the Declaration of Helsinki (22). All participants provided written informed consent.

Inclusion criteria were as follows: participants with complete data from demographic surveillance, laboratory measurements, ophthalmic examination, and questionnaires. Exclusion criteria were: significant media opacity, any type of coexisting neuro-ophthalmic disease other than diabetes-related neuropathy, refractive error of more than spherical equivalent (SE) +5 or SE -8 diopters in at least one eye, axial length (AL) > 26 mm, history of glaucoma, intraocular pressure outside the normal range, pigment epithelial detachment, macular edema, subretinal or intraretinal fluid, epiretinal membrane, uveitis or other retinal diseases, diagnosis with type 1 diabetes, or any history of ocular surgery (laser photocoagulation, intravitreal injection, and vitrectomy). Recruited participants were categorized as control individuals without T2DM, diabetic individuals with no DR, and diabetic individuals with DR, evaluated by two masked ophthalmologists based on color fundus images. DR was classified based on the presence of mild, moderate or severe non-proliferative diabetic retinopathy.

### 2.2 Ophthalmic examination

All examinations were performed at the community hospital or residential committee to which the respondents belonged. The protocol included comprehensive clinical assessments, questionnaires, and provision of blood samples for laboratory assays, which were administered by doctors, optometrists and nurses. Clinical investigations included assessment of visual acuity, optometric assays, slit-lamp examination, intraocular pressure, axial length, mydriasis, direct ophthalmoscopy of the posterior segment, fundus photography, ultra-wide field retinal imaging, swept-source optical coherence tomography (SS-OCT) and optical coherence tomography angiography (OCTA).

### 2.3 Fundus photography and DR grading system

Color fundus photographs with two fields (optic disc-centered and fovea-centered) were obtained using a stereoscopic fundus camera (Nonmyd WX3D, Kowa Company Ltd., Japan) after dilatation of both eyes on the basis of Early Treatment of Diabetic Retinopathy (ETDRS). The severity of DR in each eye was graded according to the International DR Classification System. With progressively increasing risk of retinopathy, DR was classified as no apparent retinopathy, mild NPDR, moderate NPDR, severe NPDR and proliferative diabetic retinopathy (PDR). Individuals with DR in either eye were defined as patients with DR.

### 2.4 SS-OCT imaging

**Figure 1:**
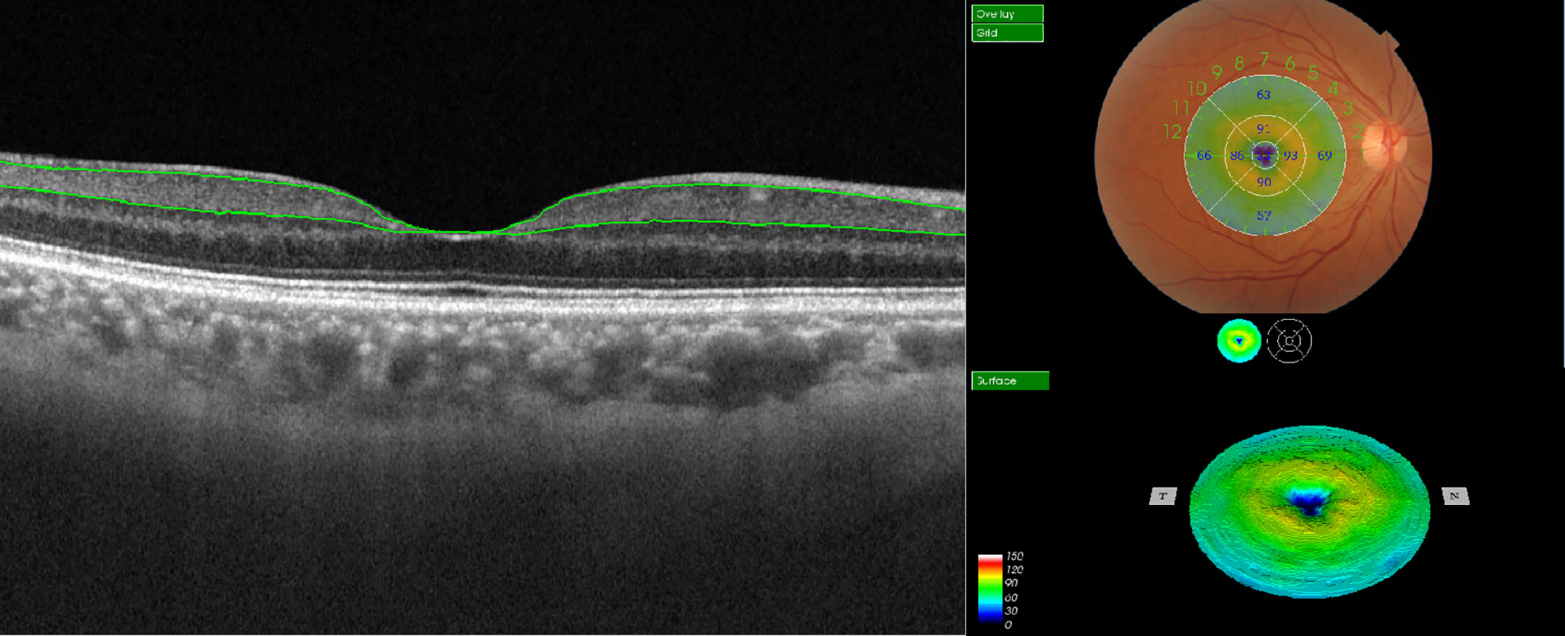
Illustration of macular ganglion cell-inner plexiform layer (mGCIPL) thickness measurement in EDTRS 9 sectors using SS-OCT. The upper green line indicates the boundary between the retinal nerve fiber layer (RNFL) and ganglion cell layer (GCL) and the lower green line highlights the boundary between the inner plexiform layer (IPL) and inner nuclear layer (INL). The individual grids are referred to as the central field, inner superior, inner nasal, inner inferior, inner temporal, outer superior, outer nasal, outer inferior, and outer temporal regions.

SS-OCT (DRI OCT Triton; Topcon Corporation, Tokyo, Japan) images of the bilateral macula were obtained after dilation. The device uses a central wavelength of 1050 nm and acquires 100,000 A-scans per second scan. The following scan patterns were performed: linear B-scan (12 mm in length) centered on the fovea at 0°; 3D macula map covering a central area of a 7 mm × 7 mm scan mode to image the retina during a 1.3-second scan time, with a scan density of 512 A-scans × 512 B-scans. The built-in software of SS-OCT could automatically identify the outer boundary of the RNFL and the outer boundary of the inner plexiform layer (IPL). Thickness of mGCIPL was determined by the distance between RNFL and IPL outer boundary segmentation. The segmented retinal thickness map included the central 1 mm region, along with the four quadrants of the inner annular ring (1-3 mm radius) and four quadrants of the outer annular ring (3-6 mm radius). The intermediate and outer rings were divided into quadrants by two intersecting lines and the thickness of each zone separately measured (inner superior, inner nasal, inner inferior, inner temporal, outer superior, outer nasal, outer inferior, and outer temporal zones; **Figure 1**). The average mGCIPL thickness from the nine grids was calculated automatically.

The same trained technician performed all OCT scans without any knowledge of patient diagnosis. The results of the automatic segmentation were evaluated by image experts and manually adjusted in case of segmentation errors. Participant data were blinded during image processing. High-quality OCT scans were included in the analysis. Scans with poor signals, incorrect algorithms, presence of pathology, blink or residual motion artifacts, and poor focus were excluded.

### 2.5 Other examinations

A comprehensive questionnaire including demographic and socioeconomic data, medical and family history, and health-related behaviors was used by the main investigator during face-to-face interviews. History collection included information on hypertension, diabetes, cardiovascular events, kidney disease, drug allergies, history of ocular disease, smoking and alcohol drinking habits, and current medication use. Blood pressure was measured twice at 5-minute intervals, and in cases where the difference between two measurements was more than 5 mmHg (diastolic) or over 10 mmHg (systolic), a third measurement was taken. The two closest measurements were averaged and recorded for analysis. Both height and body weight of participates were measured. BMI status was classified as follows: low/healthy (<18.0 to 24.9), overweight (25.0-30.0), and obese (>30.0). Waist circumference was additionally recorded. Venous blood samples (15ml) were obtained from each participant in the morning after a 12-hour overnight fast. Total cholesterol (TC), triglyceride (TG), high-density lipoprotein cholesterol (HDL-C), low-density lipoprotein cholesterol (LDL-C), glucose concentrations, and other routine blood indices were tested by using an automatic blood analyzer. Glycated hemoglobin A1c (HbA1c) was measured in participants with a self-reported history of diabetes. If the fasting blood glucose level was higher than 6.1 mmol/L in individuals without a history of diabetes, measurement of HbA1c during the second visit was recommended. Diabetes was defined as follows: (1) known history of diabetes mellitus, (2) fasting plasma glucose ≥7.0 mmol/L, (3) HbA1c ≥6.5%, or (4) current use of anti-diabetic medications.

### 2.6 Assessment and definitions of major risk factors

Glaucoma was defined according to the International Society of Geographical and Epidemiological Ophthalmology (ISGEO) criteria (23). The presence of cataract was defined in cases with nuclear, cortical, or posterior subcapsular cataracts in at least one eye based on Lens Opacities Classification System III grading (24). Diabetes duration was defined as the period between the first diagnosis or drug prescription and time of enrollment. Family history of diabetes was defined as diagnosis or type 2 diabetes in first-degree relatives. Smokers were self-reported and defined as individuals with a smoking history. Non-drinkers were defined as those who never consumed alcohol or had less than one drink per month, All other individuals were classified as a drinkers. Based on the questionnaire, physical activity was defined as moderate or intensive leisure time physical activity more than once a week. Cardiovascular events were defined as a participant self-reported history of transient ischemic attack, unstable angina pectoris, myocardial infarction, or stroke. Kidney disease was defined as a self-reported history of physician-diagnosed hematuria, albuminuria, or renal insufficiency. Hypertension was defined as blood pressure >140/90 mmHg, or receiving antihypertensive therapy. Dyslipidemia was defined in cases with at least one of the following abnormalities: elevated TC or TG, low HDL-C, elevated LDL-C, and/or hypolipidemic treatment.

### 2.7 Statistical analysis

Statistical analysis was conducted using the SPSS 23.0 statistical software package (SPSS Inc.). Continuous variables were expressed as mean (SD) and categorical variables as proportions. The included data were assessed for normality. The Chi-square test and one-way analysis of variance (ANOVA) were performed where appropriate. Mean values of axial length, intraocular pressure and mGCIPL thickness of both eyes were used for analysis. Missing data were categorized as missing at random and excluded from analysis. Confounding factors were selected in case of statistical differences *via* univariable analysis at baseline.

### 2.8 DR status

Control individuals and diabetic individuals with no DR were compared to diabetic individuals with DR. Risk factors associated with the odds of developing DR were investigated with univariable and multivariable logistic regression analysis. Results were interpreted as odds ratio (OR) and 95% confidence interval (CI). Variables with significant differences in univariable analyses were controlled as covariates.

### 2.9 mGCIPL thickness in diabetic individuals with no DR

A multivariable model was generated to analyze the associations of multiple risk factors with retinal neurodegeneration in diabetic individuals with no DR. Significant variables in the univariable linear analysis was selected. Subsequently, a multivariable linear regression analysis was performed to explore the relationship between mGCIPL thickness and risk factors.

## 3 Results

### 3.1 Participants

**Figure 2:**
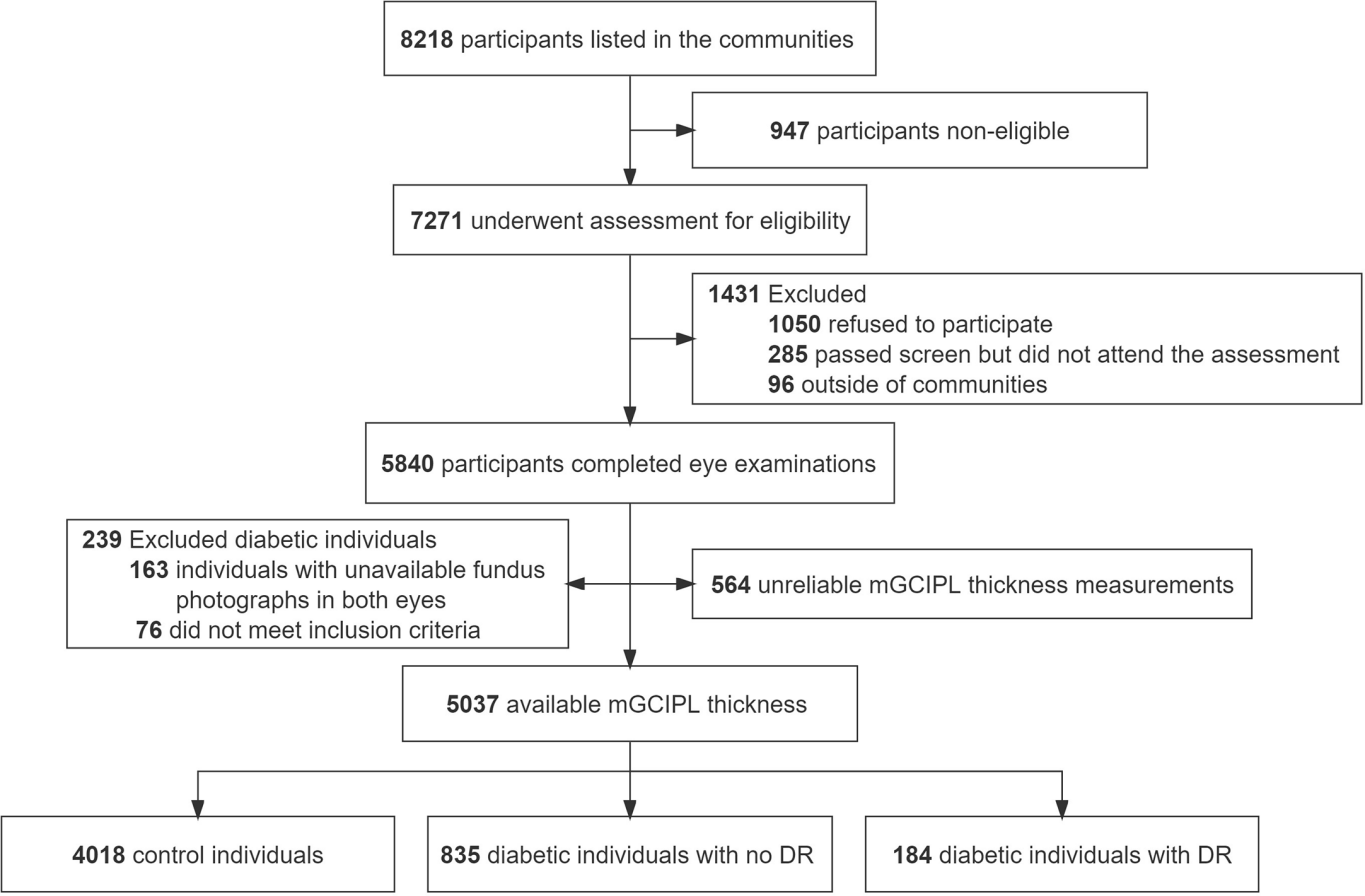
Flowchart of study participants. mGCIPL, macular ganglion cell-inner plexiform layer; DR, diabetic retinopathy.

A total of 5840 individuals were initially included, providing a 80.3% response rate. Finally, 5037 participants (3258 women [64.6%]; mean [SD] age, 62.6 [6.7] years) were evaluated, 1373 of whom were diabetic patients. All participates were categorized into three groups, specifically, 4018 control individuals, 835 diabetic individuals with no DR, and 184 diabetic individuals with DR (**Figure 2**). The basic features of the study participants were presented in **Table 1**. Statistically significant differences were detected in demographic data, cardiometabolic risk factors, laboratory investigations, and medications at enrollment among the three groups, with the exception of physical activity (*P* = 0.51), diastolic blood pressure (*P* = 0.61), and kidney disease (*P* = 0.05). Moreover, mGCIPL thickness in diabetic individuals with no DR was significantly different from that in control individuals (*P* = 0.03), but not the DR group (*P* = 0.18).

**Table 1:** Baseline cohort characteristics.

Parameters	Control individuals	No DR	DR	*P*
Demographic data				
Participants (n)	4018	835	184	
Age, mean (SD), y	62.4 (6.8)	63.2 (6.4)	63.3 (6.2)	**<0.001**
Gender, male	1368 (34.0%)	321 (38.4%)	90 (48.9%)	**<0.001**
Diabetes duration, mean (SD), y	NA	7.3 (5.7)	14.4 (7.3)	**<0.001**
Family history of diabetes	827 (20.5%)	360 (43.1%)	99 (53.8%)	**<0.001**
Smoking history				
Non-smoker	2981 (74.2%)	610 (73.1%)	124 (67.4%)	**<0.001**
Smoker	1037 (25.8%)	225 (26.9%)	60 (32.6%)	
Use of alcohol				
Non-drinker	3261 (81.2%)	664 (79.5%)	139 (75.5%)	**<0.001**
Drinker	757 (18.8%)	171 (20.5%)	45 (24.5%)	
Physical activity	2844 (70.7%)	592 (70.8%)	123 (66.8%)	0.51
Cardiometabolic risk factors				
BMI, mean (SD), kg/m2	26.0 (3.7)	26.9 (3.5)	26.8 (3.2)	**<0.001**
Waist circumference, mean (SD), cm	92.5 (10.2)	95.6 (9.5)	97.0 (9.2)	**<0.001**
Systolic blood pressure, mean (SD), mm Hg	139.0 (19.3)	144.0 (18.6)	146.9 (19.6)	**<0.001**
Diastolic blood pressure, mean (SD), mm Hg	84.9 (12.7)	84.9 (10.8)	84.1 (10.5)	0.61
Hypertension	1663 (41.3%)	523 (62.6%)	131 (71.2%)	**<0.001**
Dyslipidemia	1305 (32.4%)	381 (45.6%)	71 (38.6%)	**<0.001**
Cardiovascular events	730 (18.2%)	250 (29.9%)	58 (31.5%)	**<0.001**
Kidney disease	56 (1.4%)	20 (2.4%)	5 (2.7%)	0.05
Laboratory investigations				
Fasting plasma glucose, mean (SD), mmol/L	4.58 (0.61)	6.76 (1.91)	7.92 (2.60)	**<0.001**
HbA1c, mean (SD), %	NA	7.14 (1.15)	7.94 (1.34)	**<0.001**
Total cholesterol, mean (SD), mmol/L	5.31 (1.01)	5.20 (1.41)	5.17 (1.21)	**<0.001**
HDL cholesterol, mean (SD), mmol/L	1.17 (0.28)	1.07 (0.25)	1.06 (0.24)	**<0.001**
LDL cholesterol,mean (SD), mmol/L	3.02 (0.78)	2.94 (0.86)	2.92 (0.82)	0.01
Triglyceride, mean (SD), mmol/L	1.57 (0.87)	1.96 (1.51)	1.85 (1.41)	**<0.001**
Medications at enrolment				
Insulin	NA	114 (13.6%)	85 (46.1%)	**<0.001**
Oral antidiabetic drugs	NA	528 (63.2%)	153 (83.1%)	**<0.001**
Statins	249 (6.1%)	103 (12.3%)	20 (10.8%)	**<0.001**
Antihypertensive drugs	1268 (31.5%)	424 (50.7%)	104 (56.5%)	**<0.001**
Axial length, mean (SD), mm	23.09 (0.87)	23.04 (0.84)	22.95 (0.78)	**0.04**
Intraocular pressure, mean (SD), mm Hg	16.7 (3.3)	17.2 (3.5)	16.8 (3.5)	**0.01**
mGCIPL, mean (SD), μm	70.53 (5.77)^a^	69.92 (5.45)^b^	69.02 (5.63)^c^	**<0.001**

Significant differences in bold. ^a^ Control individuals vs No DR, P = 0.03; ^b^ No DR vs DR, P = 0.18; ^c^ Control individuals vs DR, P = 0.003; Abbreviations: BMI, body mass index (calculated as weight in kilograms divided by height in meters squared); HbA1c, hemoglobin A1c; HDL, high-density lipoprotein; LDL, low-density lipoprotein; mGCIPL, macular ganglion cell-inner plexiform layer; DR, diabetic retinopathy; NA, not applicable.

### 3.2 Odds of DR status

**Figure 3:**
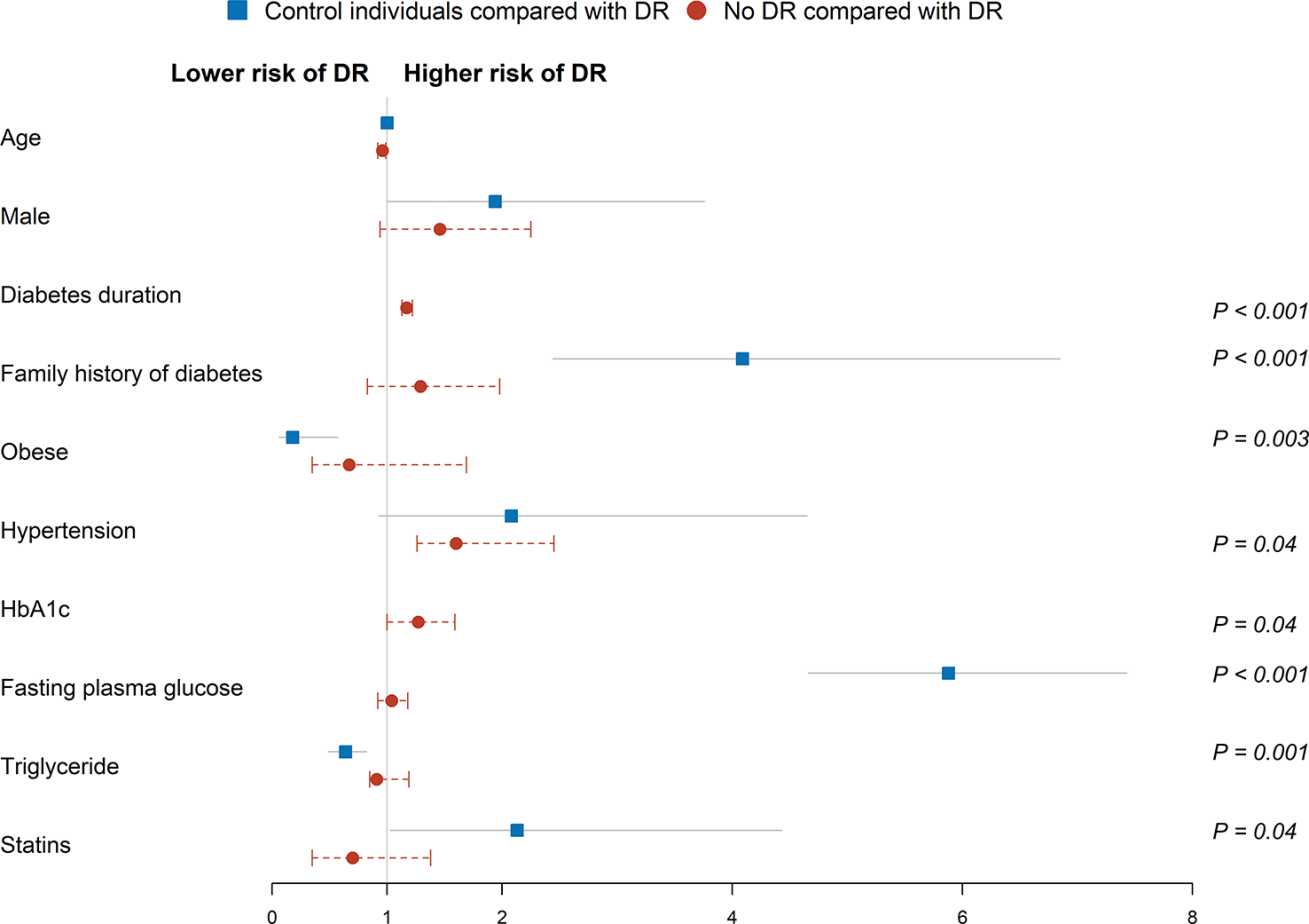
Multivariable logistic regression analysis of factors associated risk of DR diagnosis. Squares and circles represent odds ratios; whiskers represent 95% CI. Analysis was adjusted for age, gender, family history of diabetes, smoker status, overweight, waist circumference, systolic blood pressure, hypertension, cardiovascular events, fasting plasma glucose, HDL cholesterol, triglyceride, statins, antihypertensive drugs, and axial length in control individuals. Analysis was adjusted for age, gender, diabetes duration, family history of diabetes, hypertension, fasting plasma glucose, HbA1c, insulin, and oral antidiabetic drugs in the no DR group. Reference groups include age (50-59 years), female, no family history of diabetes, BMI (low/normal weight), no hypertension, no cardiovascular events, and no statins.

To determine the odds of DR diagnosis, control individuals and no DR group were individually compared with DR group *via* univariable and multivariable logistic regression analyses (**Table 2**; **Figure 3**). In univariable logistic regression analysis, male, family history of diabetes, hypertension, and high fasting plasma glucose levels displayed significant differences in terms of DR status in both control individuals and no DR group compared with the DR group. Age (60-69 years) (odds ratio [OR] 1.43, 95% confidence interval [CI] 1.01-2.02, *P* = 0.04), smoker (OR 1.39, 95% CI 1.01-1.91, *P* = 0.04), overweight (OR 1.68, 95% CI 1.18-2.41, *P* = 0.004), waist circumference (OR 1.05, 95% CI 1.03-1.06, *P* < 0.001), systolic blood pressure (OR 1.02, 95% CI 1.01-1.03, *P* < 0.001), cardiovascular events (OR 2.07, 95% CI 1.50-2.86, *P* < 0.001), HDL cholesterol (OR 0.18, 95% CI 0.10-0.34, *P* < 0.001), triglyceride (OR 1.28, 95% CI 1.13-1.44, *P* < 0.001), statins (OR 1.85, 95% CI 1.14-2.99, *P* = 0.01), antihypertensive drugs (OR 2.82, 95% CI 2.09 to 3.80, *P* < 0.001), and axial length (OR 0.83, 95% CI 0.69-0.99, *P* = 0.04) were associated with DR status compared to control individuals. In addition, diabetes duration (OR 1.17, 95% CI 1.14-1.21, *P* < 0.001), HbA1c (OR 1.62, 95% CI 1.41-1.85, *P* < 0.001), insulin (OR 4.43, 95% CI 3.82-5.71, *P* < 0.001), and oral antidiabetic drugs (OR 3.11, 95% CI 2.00-4.83, *P* < 0.001) were associated with diagnosis of DR compared to the no DR group.

**Table 2:** Univariable and multivariable logistic regression-odds of DR status.

Parameters	Univariable logistic regression						Multivariable logistic regression					
	Control individuals compared with DR			No DR compared with DR			Control individuals compared with DR			No DR compared with DR		
Demographic data	OR	95% CI	*p*-value	OR	95% CI	*p*-value	aOR	95% CI	*p*-value	aOR	95% CI	*p*-value
Age per unit increase	1.02	1.00 to 1.04	0.08	1.00	0.97 to 1.03	0.90	1.00	0.96 to 1.04	0.98	0.96	0.92 to 0.99	0.41
50-59 years	NA	NA	NA	NA	NA	NA	NA	NA	NA	NA	NA	NA
60-69 years	1.43	1.01 to 2.02	**0.04**	1.10	0.76 to 1.60	0.60	0.70	0.42 to 1.16	0.17	0.79	0.48 to 1.31	0.36
≥70 years	1.56	0.99 to 2.44	0.05	1.15	0.71 to 1.86	0.58	1.97	0.82 to 4.73	0.13	0.46	0.24 to 0.91	0.24
Gender												
Female	NA	NA	NA	NA	NA	NA	NA	NA	NA	NA	NA	NA
Male	1.86	1.38 to 2.49	**<0.001**	1.53	1.11 to 2.11	**0.01**	1.94	1.00 to 3.76	0.05	1.46	0.94 to 2.25	0.09
Diabetes duration	NA	NA	NA	1.17	1.14 to 1.21	**<0.001**	NA	NA	NA	1.17	1.13 to 1.22	**<0.001**
Family history of diabetes	4.49	3.33 to 6.07	**<0.001**	1.54	1.12 to 2.12	**0.01**	4.09	2.44 to 6.85	**<0.001**	1.29	0.83 to 1.98	0.26
Smoking history												
Non-smoker	NA	NA	NA	NA	NA	NA	NA	NA	NA	NA	NA	NA
Smoker	1.39	1.01 to 1.91	**0.04**	1.31	0.93 to 1.85	0.12	1.20	0.63 to 2.27	0.59	1.21	0.69 to 2.11	0.51
Use of alcohol												
Non-drinker	NA	NA	NA	NA	NA	NA	NA	NA	NA	NA	NA	NA
Drinker	1.40	0.99 to 1.97	0.06	1.26	0.86 to 1.83	0.23	1.15	0.58 to 2.28	0.70	0.67	0.37 to 1.20	0.18
Physical activity	0.83	0.61 to 1.14	0.25	0.83	0.59 to 1.16	0.28	0.89	0.52 to 1.53	0.68	0.76	0.47 to 1.23	0.26
Cardiometabolic risk factors												
BMI group												
Low/healthy (<18.0 to 24.9)	NA	NA	NA	NA	NA	NA	NA	NA	NA	NA	NA	NA
Overweight (25.0-30.0)	1.68	1.18 to 2.41	**0.004**	1.06	0.72 to 1.56	0.76	0.87	0.52 to 1.44	0.58	1.00	0.59 to 1.69	0.98
Obese (>30.0)	1.58	0.95 to 2.61	0.08	0.89	0.52 to 1.52	0.66	0.18	0.06 to 0.57	**0.003**	0.67	0.35 to 1.69	0.52
Waist circumference (cm)	1.05	1.03 to 1.06	**<0.001**	1.02	1.00 to 1.04	0.08	1.00	0.98 to 1.03	0.42	1.01	0.98 to 1.04	0.56
Systolic blood pressure (mm Hg)	1.02	1.01 to 1.03	**<0.001**	1.01	1.00 to 1.02	0.07	1.01	1.00 to 1.03	0.13	1.01	1.00 to 1.03	0.06
Diastolic blood pressure (mm Hg)	0.99	0.98 to 1.01	0.36	0.99	0.98 to 1.01	0.37	0.95	0.92 to 0.98	0.11	1.02	1.00 to 1.04	0.12
Hypertension	3.50	2.53 to 4.85	**<0.001**	1.48	1.04 to 2.09	**0.03**	2.08	0.93 to 4.65	0.07	1.60	1.26 to 2.45	**0.04**
Dyslipidemia	1.31	0.96 to 1.77	0.09	0.75	0.54 to 1.04	0.08	0.95	0.48 to 1.88	0.87	0.65	0.42 to 1.01	0.06
Cardiovascular events	2.07	1.50 to 2.86	**<0.001**	1.08	0.76 to 1.52	0.67	1.51	0.85 to 2.67	0.16	0.85	0.53 to 1.36	0.51
Kidney disease	1.98	0.78 to 4.99	0.15	1.14	0.42 to 3.07	0.80	1.54	0.32 to 7.36	0.59	0.64	0.20 to 2.01	0.44
Laboratory investigations												
Fasting plasma glucose (mmol/L)	6.45	5.25 to 7.92	**<0.001**	1.25	1.17 to 1.34	**<0.001**	5.88	4.66 to 7.43	**<0.001**	1.04	0.92 to 1.18	0.53
HbA1c (%)	NA	NA	NA	1.62	1.41 to 1.85	**<0.001**	NA	NA	NA	1.27	1.00 to 1.59	**0.04**
Total cholesterol (mmol/L)	0.87	0.75 to 1.01	0.06	0.97	0.85 to 1.12	0.70	0.98	0.74 to 1.31	0.98	1.02	0.85 to 1.22	0.88
HDL cholesterol (mmol/L)	0.18	0.10 to 0.34	**<0.001**	0.80	0.41 to 1.54	0.51	0.35	0.11 to 1.14	0.08	2.00	0.80 to 5.01	0.14
LDL cholesterol (mmol/L)	0.84	0.69 to 1.01	0.07	0.96	0.80 to 1.16	0.68	0.84	0.61 to 1.17	0.31	1.04	0.81 to 1.34	0.74
Triglyceride (mmol/L)	1.28	1.13 to 1.44	**<0.001**	0.95	0.84 to 1.07	0.37	0.64	0.49 to 0.82	**0.001**	0.91	0.85 to 1.19	0.95
Medications at enrolment												
Insulin	NA	NA	NA	4.43	3.82 to 5.71	**<0.001**	NA	NA	NA	1.67	1.04 to 2.68	0.14
Oral antidiabetic drugs	NA	NA	NA	3.11	2.00 to 4.83	**<0.001**	NA	NA	NA	0.78	0.41 to 1.48	0.44
Statins	1.85	1.14 to 2.99	0.01	0.87	0.52 to 1.44	0.58	2.13	1.03 to 4.43	**0.04**	0.70	0.35 to 1.38	0.30
Antihypertensive drugs	2.82	2.09 to 3.80	**<0.001**	1.26	0.91 to 1.74	0.16	1.30	0.63 to 2.68	0.47	0.56	0.28 to 1.09	0.09
Axial length, mm	0.83	0.69 to 0.99	**0.04**	0.88	0.72 to 1.08	0.22	0.72	0.53 to 0.99	0.05	0.85	0.64 to 1.14	0.28
Intraocular pressure, mm Hg	1.00	0.96 to 1.05	0.92	0.96	0.92 to 1.01	0.11	0.91	0.84 to 0.99	0.32	0.95	0.89 to 1.02	0.19

Significant differences in bold. Abbreviations: BMI, body mass index (calculated as weight in kilograms divided by height in meters squared); HbA1c, hemoglobin A1c; HDL, high-density lipoprotein; LDL, low-density lipoprotein; DR, diabetic retinopathy; OR, odds ratio; CI, confidence interval; aOR, adjusted odds ratio; NA, not applicable; Reference groups include age (50-59 years), female, no family history of diabetes, non-smoker, non-drinker, low/healthy (<18.0 to 24.9), no physical activity, no hypertension, no dyslipidemia, no insulin, no oral antidiabetic drugs, no statins, and no antihypertensive drugs.

Multivariable logistic regression analysis was performed after adjusting for significant covariates. Family history of diabetes (adjusted odds ratio [aOR] 4.09, 95% CI 2.44-6.85, *P* < 0.001), obese ([aOR] 0.18, 95% CI 0.06-0.57, *P* = 0.003), fasting plasma glucose ([aOR] 5.88, 95% CI 4.66-7.43, *P* < 0.001), triglyceride ([aOR] 0.64, 95% CI 0.49-0.82, *P =* 0.001) and statins ([aOR] 2.13, 95% CI 1.03-4.43, *P* = 0.04) were associated with DR status in the control individuals. Similarly, diabetes duration ([aOR] 1.17, 95% CI 1.13-1.22, *P* < 0.001), hypertension ([aOR] 1.60, 95% CI 1.26-2.45, *P* = 0.04), and HbA1c ([aOR] 1.27, 95% CI 1.00-1.59, *P* = 0.04) were associated with higher odds of DR in the no DR group.

### 3.3 mGCIPL thickness in diabetic individuals with no DR

In univariable analysis, age per unit increase (β = -0.22 [95% CI, -0.28 to -0.17] μm; *P* < 0.001), 60-69 years (β = -1.58 [95% CI, -2.40 to -0.75] μm; *P* < 0.001), ≥70 years (β = -3.80 [95% CI, -4.90 to -2.71] μm; *P* < 0.001), male (β = -0.83 [95% CI, -1.59 to -0.07] μm; *P* = 0.03), waist circumference (β = -0.06 [95% CI, -0.10 to -0.02] μm; *P* = 0.01), hypertension (β = -0.97 [95% CI, -1.73 to -0.20] μm; *P* = 0.01), cardiovascular events (β = -1.18 [95% CI, -1.99 to -0.38] μm; *P* = 0.004), and axial length (β = -1.00 [95% CI, -1.44 to -0.55] μm; *P* < 0.001) were correlated with thinning of mGCIPL. However, total cholesterol (β = 0.39 [95% CI, 0.06 to 0.71] μm; *P* = 0.02) and LDL cholesterol (β = 0.45 [95% CI, 0.02 to 0.88] μm; *P* = 0.04) were positively associated with mGCIPL thickness.

**Figure 4:**
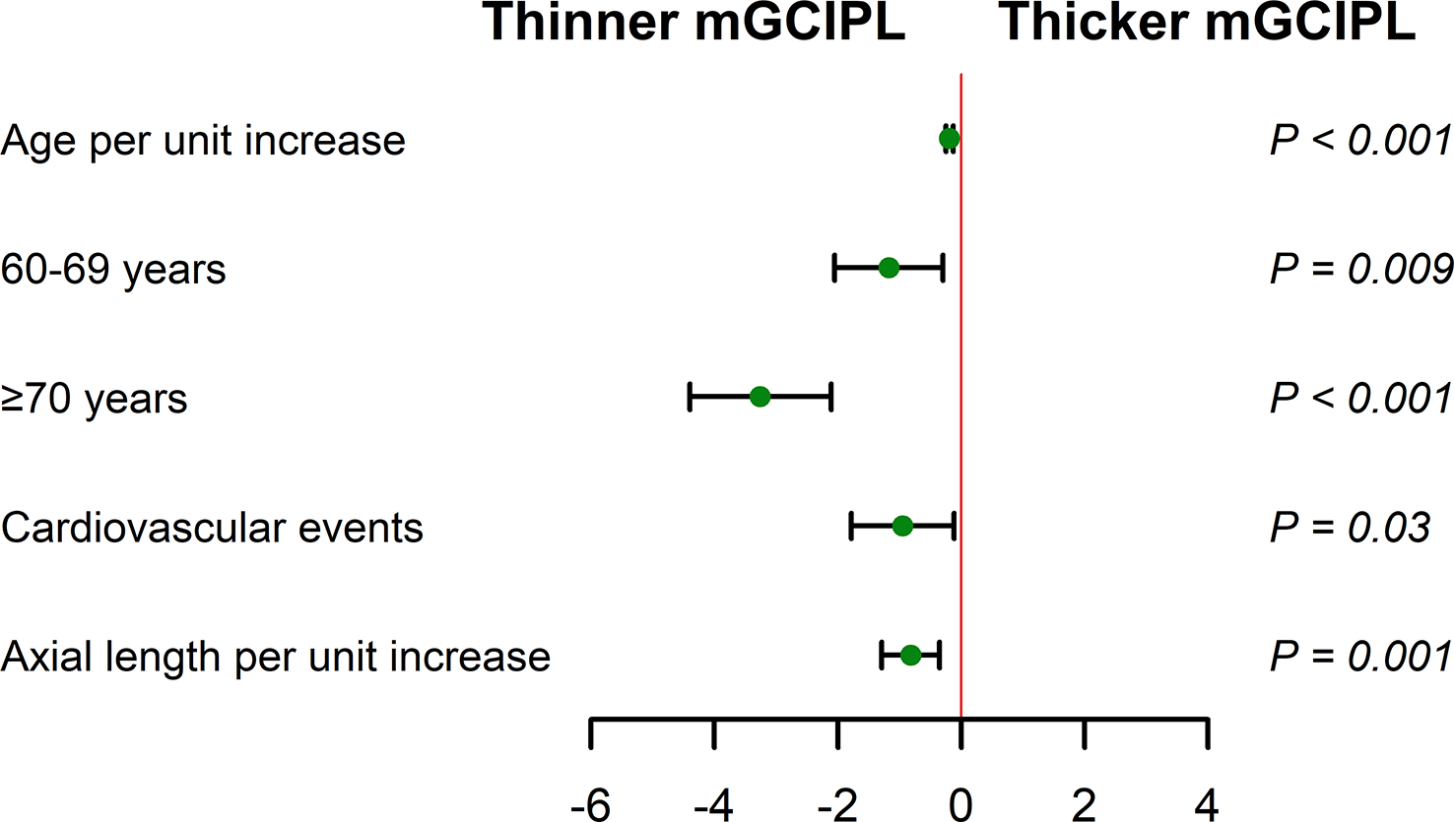
Multivariable linear regression analysis of associations of mGCIPL thickness with age, cardiovascular events, and axial length (adjusted for age, gender, waist circumference, hypertension, cardiovascular events, total cholesterol, LDL cholesterol, and axial length) in diabetic individuals with no DR. Reference groups include age (50-59 years) and no cardiovascular events. Whiskers represent range of mGCIPL thickness. .

Multivariable linear regression analysis revealed correlations of age per unit increase (adjusted β = -0.19 [95% CI, -0.25 to -0.13] μm; *P* < 0.001), 60-69 years (β = -1.17 [95% CI, -2.05 to -0.30] μm; *P* = 0.001), ≥70 years (β = -3.26 [95% CI, -4.40 to -2.11] μm; *P* < 0.001), cardiovascular events (β = -0.95 [95% CI, -1.78 to -0.12] μm; *P* = 0.03), and axial length per unit increase (β = -0.82 [95% CI, -1.29 to -0.35] μm; *P* = 0.001) with thinning of mGCIPL after adjusting for significant covariates (**Table 3**; **Figure 4**). However, no significant associations were observed with male, waist circumference, hypertension, TC, and LDL cholesterol.

**Table 3:** univariable and multivariable linear regression investigating associations with mGCIPL thickness in diabetic individuals with no DR.

	Univariable analysis			Multivariable analysis		
Parameters	β	95% CI	p-value	β	95% CI	p-value
Demographic data						
Age per unit increase	-0.22	-0.28 to -0.17	**<0.001**	-0.19	-0.25 to -0.13	**<0.001**
50-59 years	NA	NA	NA	NA	NA	NA
60-69 years	-1.58	-2.40 to -0.75	**<0.001**	-1.17	-2.05 to -0.30	**0.009**
≥70 years	-3.80	-4.90 to -2.71	**<0.001**	-3.26	-4.40 to -2.11	**<0.001**
Male	-0.83	-1.59 to -0.07	**0.03**	0.14	-0.71 to 0.99	0.75
Diabetes duration	-0.05	-0.12 to 0.03	0.21	0.02	-0.06 to 0.09	0.62
Family history of diabetes	-0.08	-0.83 to 0.67	0.84	-0.37	-1.13 to 0.39	0.35
Smoking history						
Non-smoker	NA	NA	NA	NA	NA	NA
Smoker	-0.25	-1.09 to 0.59	0.56	0.00	-1.01 to 1.00	0.99
Use of alcohol						
Non-drinker	NA	NA	NA	NA	NA	NA
Drinker	0.57	-0.35 to 1.49	0.22	0.80	-0.30 to 1.89	0.16
Physical activity	0.11	-0.70 to 0.93	0.79	0.28	-0.54 to 1.09	0.50
Cardiometabolic risk factors						
BMI	-0.05	-0.16 to 0.06	0.34	-0.01	-0.11 to 0.10	0.93
Low/healthy (<18.0 to 24.9)	NA	NA	NA	NA	NA	NA
Overweight (25.0-30.0)	-0.56	-1.30 to 0.18	0.14	-0.35	-1.15 to 0.45	0.40
Obese (>30.0)	-0.81	-1.85 to 0.24	0.13	-0.43	-1.50 to 0.65	0.44
Waist circumference (cm)	-0.06	-0.10 to -0.02	**0.01**	-0.03	-0.07 to 0.01	0.14
Systolic blood pressure (mm Hg)	-0.01	-0.03 to 0.01	0.21	0.00	-0.02 to 0.02	0.95
Diastolic blood pressure (mm Hg)	0.01	-0.02 to 0.05	0.54	-0.02	-0.06 to 0.02	0.35
Hypertension	-0.97	-1.73 to -0.20	**0.01**	-0.26	-1.06 to 0.55	0.53
Dyslipidemia	0.38	-0.36 to 1.13	0.32	0.52	-0.27 to 1.31	0.20
Cardiovascular events	-1.18	-1.99 to -0.38	**0.004**	-0.95	-1.78 to -0.12	**0.03**
Kidney disease	-1.04	-3.46 to 1.39	0.40	-1.20	-3.63 to 1.23	0.33
Laboratory investigations						
Fasting plasma glucose (mmol/L)	-0.04	-0.24 to 0.15	0.66	-0.10	-0.30 to 0.11	0.35
HbA1c (%)	-0.28	-0.62 to 0.07	0.11	-0.28	-0.63 to 0.07	0.12
Total cholesterol (mmol/L)	0.39	0.06 to 0.71	**0.02**	0.18	-0.53 to 0.89	0.62
HDL cholesterol (mmol/L)	1.21	0.28 to 2.70	0.11	-0.41	-2.01 to 1.19	0.61
LDL cholesterol (mmol/L)	0.45	0.02 to 0.88	**0.04**	0.10	-0.84 to 1.04	0.84
Triglyceride (mmol/L)	-0.02	-0.27 to 0.22	0.85	-0.04	-0.38 to 0.31	0.83
Medications at enrolment						
Insulin	-0.66	-1.74 to 0.42	0.23	-0.23	-1.30 to 0.84	0.68
Oral antidiabetic drugs	-0.69	-1.48 to 0.11	0.09	-0.25	-1.05 to 0.55	0.54
Statins	0.61	-0.52 to 1.73	0.29	0.53	-0.51 to 1.56	0.32
Antihypertensive drugs	-0.32	-1.06 to 0.42	0.39	0.93	-0.21 to 2.07	0.11
Axial length per unit increase	-1.00	-1.44 to -0.55	**<0.001**	-0.82	-1.29 to -0.35	**0.001**
IOP per unit increase	-0.05	-0.15 to 0.06	0.42	-0.11	-0.22 to 0.00	0.05

Significant differences in bold. Abbreviations: mGCIPL, macular ganglion cell-inner plexiform layer; DR, diabetic retinopathy; BMI, body mass index (calculated as weight in kilograms divided by height in meters squared); HbA1c, hemoglobin A1c; HDL, high-density lipoprotein; LDL, low-density lipoprotein; IOP, intraocular pressure; β, regression coefficient; CI, confidence interval; NA, not applicable.

## 4 Discussion

In this cross-sectional study, we found that in contrast to BMI and triglyceride, family history of diabetes, fasting plasma glucose, and statins were associated with the increased odds of DR diagnosis in the general population. Diabetes duration, hypertension, and HbA1c were identified as risk factors for DR formation in diabetic patients. Cardiovascular events and axial length were not correlated with DR diagnosis but negatively associated with mGCIPL thickness. Moreover, mGCIPL thickness showed a negative correlation with increasing age.

The odds of higher DR status were associated with multiple risk factors in this study. Family history of diabetes increased the risk of diabetes mellitus and prevalence of DR in healthy young adults (25), and is considered a promising health tool for evaluating the risk of T2DM and DR (25, 26). Notably, an earlier cross-sectional clinic-based study showed reduced risk of DR with higher BMI (27). Consistent with this finding, high BMI levels were correlated with decreased risk of DR in our study. The use of statins was additionally related to DR status in the control individuals but not no DR group in our study. In earlier reports, statins therapy increased the risk of new-onset diabetes (28), which was speculated to reduce insulin production or increase insulin resistance and thereby affect glucose homeostasis (29). However, the association of dyslipidemia and statins treatment with diabetic retinopathy remains controversial (30). The current investigation presented an association of triglyceride with DR in control individuals but not the no DR group. Conversely, fasting blood glucose and 2-hour postprandial glucose along with triglyceride levels were significantly related to prevalence of DR (31). Studies to date suggest that triglyceride levels serve as an inconsistent parameter and have an overall modest association with DR (32). Fasting plasma glucose was associated with significantly increased odds of DR status in control individuals. A similar previous study reported higher blood glucose level as a risk factor for retinopathy (including DR) in non-diabetic individuals (33). Moreover, 2-hour postprandial blood glucose levels were significantly associated with progression of DR in population with no diabetes (34).

One interesting aspect of this study was the inclusion of non-diabetic control individuals to identify risk factors for DR status. To our knowledge, few studies to date have focused on risk factors for DR in older non-diabetic populations. Although diabetes was an important determinant of DR, other risk factors (including family history of diabetes, fasting plasma glucose, and statins) identified in our study should not be overlooked, since these factors are also the baseline characteristics for onset of type 2 diabetes (35). DR prevalence in newly diagnosed diabetes ranged from 2.8% to 28.6% (36–39). In addition, T2DM was confirmed in 10.53% (40) and 20% (41) of newly diagnosed patients with DR based on initial fundus examination and subsequent blood glucose testing, highlighting that a proportion of the general population lacking health awareness may have suffered from diabetes for more than 10 years before diagnosis of DR (42). Thus, elucidation of the risk factors that contribute to increasing the odds of DR status could facilitate the development of effective measures to prevent or manage the onset of DR.

Significant variations in the patterns, strength, and consistency of the major risk factors for DR have been reported among numerous epidemiological investigations (13). In this study, independent factors associated with DR in diabetic patients included diabetes duration, hypertension, and HbA1c, consistent with a systematic literature review showing increased DR prevalence with long diabetes duration, high HbA1c, and high blood pressure levels in diabetic patients (13). Diabetes duration has long been characterized as an independent risk factor for DR (43, 44), and may be considered a reflection of total glucose control and risk factor exposure over time (45). Moreover, hypertension has consistently been identified as a risk factor in the formation of DR (46). In our study, diabetic individuals with hypertension had a 1.6 times higher chances of developing DR than those without hypertension, in keeping with previous reports (47, 48). HbA1c has been identified as another common risk factor of retinopathy in DR studies (49). Previous key studies provided evidence that well-controlled HbA1c levels reduced the possibility of DR in individuals with diabetes. Moreover, risk of retinopathy could be reduced by 25-40% for every 1% decrease in HbA1c level (50, 51). Similarly, in our study, risk of retinopathy was increased by 27% for each 1% increase in HbA1c level.

DR has long been considered a microangiopathy, but accumulating experimental and clinical studies suggest that diabetic retinal neurodegeneration represents damage to the retina in diabetic patients (4, 7). In our study, the major risk factors for diabetic retinal neurodegeneration characterized by mGCIPL thickness were age, cardiovascular events, and axial length in diabetic patients with no DR. Moreover, thinning mGCIPL was independently associated with age (52). The rate of mGCIPL loss in diabetic individuals with no DR was 0.19 μm with every progressive year of age, which was higher than that recorded earlier [0.12 μm (52), 0.159 μm (53), and 0.18 μm (54)] in healthy populations from different studies. Our study further validated the relationship between cardiovascular events and retinal neurodegeneration. Cardiovascular events are thought to contribute to impaired functioning of hemodynamic autoregulation, which can predispose to ischemia and lead to higher levels of neuroinflammation that is detrimental for neuronal cells (55). Retinal neuronal cells are highly susceptible to ischemia, owning to their high energy demand, and are fully dependent on continuous nutrients supply *via* the microvasculature (56). However, large cohort studies are required for further in-depth exploration. Axial length was inversely linked to inner retinal layer thickness (57). Mean macular ganglion cell complex layer thickness was reported to decrease by about 1.56 μm per 1 mm increase in axial length (53), which was greater than the value obtained in our study (0.82 μm). However, the ganglion cell complex layer was thicker than the mGCIPL layer. Thus, the axial length should be considered when using mGCIPL thickness to evaluate retinal neurodegeneration in patients with diabetes.

The strengths of this study include application of average mGCIPL thickness from both eyes, automated quantitative measurements *via* SS-OCT, detailed assessment of risk factors, and simultaneous analysis of the association of multiple risk factors with DR status and retinal neurodegeneration in a large population-based sample. Additionally, although diabetes is a prerequisite for DR, knowledge of the correlations between other risk factors and DR status may help to shift the focus of DR prevention from diabetic patients to general individuals, thereby providing further guidance for a healthy life in the general population. As multifactorial intervention can significantly reduce cardiovascular events (58, 59), its early application may also be effective in prevention of DR.

The current study presents several limitations that should be taken into consideration. First, the cross-sectional nature of the study limited the inferences of causality. Second, sample sizes for no DR and DR groups were relatively small compared to the control group. Third, the method of identifying retinopathy *via* two fundus photographs per eye was simpler than that used in previous studies (60, 61), which may have led to underestimation of the prevalence of DR. Fourth, data on smoking, alcohol consumption, diabetes duration, family history of diabetes, cardiovascular events, and kidney disease were self-reported and not verified using medical records. Fifth, although individuals with glaucoma neuropathy were excluded from study, participants with preclinical glaucoma could not be excluded completely without visual field testing.

## 5 Conclusion

The current study provides critical population-based data on the associations of multiple risk factors with DR diagnosis and retinal neurodegeneration. The risk factors linked to DR status in diabetic patients are diabetes duration, hypertension, and HbA1c. Moreover, the risk factors contributing to increased odds of DR development in the general population, including family history of diabetes, fasting plasma glucose, and statins, should be paid further attention. As mGCIPL thickness is affected in diabetic individuals with no DR, age, cardiovascular events, and axial length, identified as potential risk factors, should be further explored. Overall, more precise understanding of the major risk factors and their associations with DR status and retinal neurodegeneration is crucial for public health education to facilitate disease management and improve patient outcomes.

## Data availability statement

The original contributions presented in the study are included in the article. Further inquiries can be directed to the corresponding authors.

## Ethics statement

The studies involving human participants were reviewed and approved by Ethical Committee of Tianjin Medical University Eye Hospital. The patients/participants provided their written informed consent to participate in this study.

## Author contributions

All authors listed have made a substantial, direct and intellectual contribution to the work, and approved it for publication.
